# The contrasting properties of conservation and correlated phylogeny in protein functional residue prediction

**DOI:** 10.1186/1471-2105-9-51

**Published:** 2008-01-25

**Authors:** Jonathan R Manning, Emily R Jefferson, Geoffrey J Barton

**Affiliations:** 1 School of Life Sciences Research, University of Dundee, UK

## Abstract

**Background:**

Amino acids responsible for structure, core function or specificity may be inferred from multiple protein sequence alignments where a limited set of residue types are tolerated. The rise in available protein sequences continues to increase the power of techniques based on this principle.

**Results:**

A new algorithm, SMERFS, for predicting protein functional sites from multiple sequences alignments was compared to 14 conservation measures and to the MINER algorithm. Validation was performed on an automatically generated dataset of 1457 families derived from the protein interactions database SNAPPI-DB, and a smaller manually curated set of 148 families. The best performing measure overall was *Williamson *property entropy, with *ROC*_0.1 _scores of 0.0087 and 0.0114 for domain and small molecule contact prediction, respectively. The *Lancet *method performed worse than random on protein-protein interaction site prediction (*ROC*_0.1 _score of 0.0008). The SMERFS algorithm gave similar accuracy to the phylogenetic tree-based MINER algorithm but was superior to *Williamson *in prediction of non-catalytic transient complex interfaces. SMERFS predicts sites that are significantly more solvent accessible compared to *Williamson*.

**Conclusion:**

*Williamson *property entropy is the the best performing of 14 conservation measures examined. The difference in performance of SMERFS relative to *Williamson *in manually defined complexes was dependent on complex type. The best choice of analysis method is therefore dependent on the system of interest. Additional computation employed by Miner in calculation of phylogenetic trees did not produce improved results over SMERFS. SMERFS performance was improved by use of windows over alignment columns, illustrating the necessity of considering the local environment of positions when assessing their functional significance.

## Background

A major focus of research in molecular biology is to determine the function of the gene products encoded in an organism's genome. Genes that code for protein are heavily studied by experimental methods, but these approaches may take years to provide a detailed understanding of a single gene's function within the context of the organism and its life-cycle. As a consequence, even though the complete genome sequences of more than 52 eukaryotes, 47 archaea, and 517 bacteria are currently known [1], and the location of protein-coding genes can be determined with reasonable accuracy [2], only a few thousand proteins have been functionally characterised with a high degree of confidence. Accordingly, computational methods to predict the function of a protein given its amino acid sequence play a major role in guiding the experimental characterisation of a newly sequenced genome. The majority of methods aim to classify protein sequences by function, and apply a range of techniques from pair-wise sequence searching to profile-profile HMMs [3] to identify similarities to well-characterised proteins annotated by the Gene Ontology [4]. Although assigning a protein to a broad functional class (e.g. hydrolase or synthase) may be achieved with a reasonable degree of confidence by these methods, reliably predicting which residues are involved in conferring specificity for a particular substrate remains a major challenge. The present work is concerned with identifying "functional residues" that might be associated either with core function (CF) or specificity (SD [5]).

**Figure 1:**
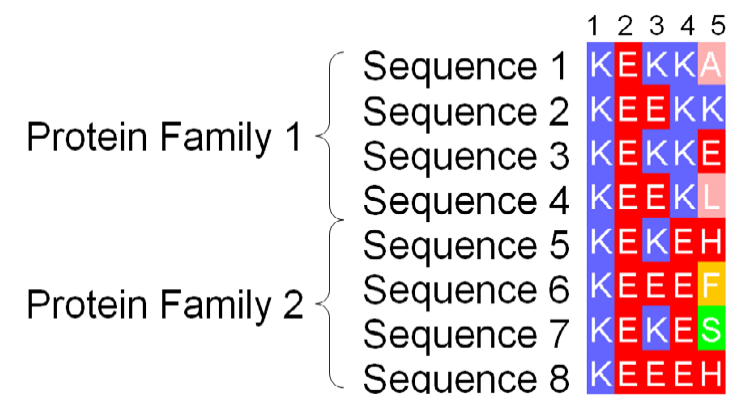
**Fragment of an Example Alignment**. Illustration of the difference between highly conserved positions likely to be responsible for core structure and/or function and specificity-defining (SD) positions of a multiple sequence alignment. Columns 1 and 2 illustrate positions crucial in all family members. Column 3 shows a similar, though less stringent global pattern of conservation. Column 4 in contrast represents an SD position, where only a single amino acid is tolerated by each subfamily. Column 5 represents a non-conserved position for comparison.

The most common approach for predicting functional residues from the amino acid sequence has been to exploit the evolutionary information present in an accurate multiple protein sequence alignment. Providing the sequences are sufficiently diverse, the location of positions in the alignment with invariant, or highly "conserved" amino acids may suggest structural or functional importance. A number of different scoring functions have been developed to quantify conservation of protein sequence alignment positions as reviewed in [6]. In recent work by Capra and Singh [7], important positions in a sequence alignment are located by differentiating amino acid distributions at positions under evolutionary pressure from those of positions that are not. All these techniques will identify columns 1–4 of the multiple alignment fragment shown in Figure 1 as conserved, but will not discriminate between columns 3 and 4. Since in a multiple sequence alignment the sequences are grouped by overall similarity, column 4 suggests that the position is important to all sequences, but conservation of Lys in one subfamily and Glu in the other indicates that the position may have a role in defining the structural or functional specificity within the protein family. A number of algorithms seek explicitly to identify such Specificity Defining (SD) positions and so discriminate between columns 3 and 4. For example, the AMAS algorithm [8] identifies SD positions that have conserved physicochemical properties within pre-defined sub-families of proteins (e.g. +ve charge), yet exhibit different properties between the sub-families (e.g. +ve charge compared to -ve). In the 'Evolutionary Trace' (ET) technique [9] alignments are first sub-grouped by the use of partitions on a phylogenetic tree, and predictions are interpreted by mapping onto protein structures. ET originally required the tree partition to be chosen manually, but subsequent developments have attempted to address this issue [10,11]. In contrast to AMAS, and ET, the 'Sequence Space' algorithm [12] which represents sequences of an alignment as vectors in high dimensional space, does not require pre-grouping of the sequences. In Sequence Space, principal components analysis is employed to derive both the principal sequence subgroupings, and the SD positions characteristic of each group. More recently, Marttinen [13] applied a Bayesian statistical approach in an analogous manner, again deriving both the optimal subgrouping and functionally relevant positions of an alignment.

The SD methods discussed thus far assume a single underlying subgrouping. In contrast, the ConSurf method [14-16] infers a phylogenetic tree to normalise an entropy score over all columns in an alignment. The method incorporates a model of substitution rates along branches of the tree and in the most recent version this is performed within a Bayesian framework [17]. Alignment positions are classified based on the normalised entropy score, and colour-coded classes mapped to the surface of an example structure. A different approach was taken in the MINER algorithm, where a phylogentic tree is derived for windows of a given size at each position in the alignment. The degree to which these trees correlate with the tree derived from the whole alignment is determined by counting topological differences. The highest correlating regions, referred to as 'phylogenetic motifs', are proposed as functionally significant [18]. The MINER and ConSurf approaches are attractive since no artificial divisions of the alignment are necessary. However, while multiple valid phylogenetic trees are possible given a single alignment-derived distance matrix, both these techniques derive only one. If phylogeny estimation is not the objective of the investigation, assuming a single phylogenetic tree unnecessarily removes information. This problem is not intractable, and probabilistic models [19] can facilitate representation of multiple possible trees. However this level of complexity may not be necessary, since the phylogentic structure of alignment columns may be compared to that of the whole by use of distance matrices without first inferring a tree.

Pazos and Valencia [20] demonstrated that distance matrices derived from single columns of protein family alignments can be effective in the prediction of protein-protein interactions. A 'mutational behavior' method was also demonstrated, showing that distance matrices generated at single alignment positions that correlate with a matrix from the whole alignment can be indicative of functional sites [21]. However, the mutational behaviour method is limited to single alignment columns which may render it susceptible to noise. The alternative explored in the SMERFS algorithm [22] introduced in this paper is to work directly from distance matrices and to consider the local neighbourhood of each position in the alignment. The output of SMERFS is somewhat similar to the similarity deviation score produced by the three-dimensional cluster analysis method of Landgraf and coworkers [23]. However unlike the method of Landgraf, SMERFS considers neighbours in sequence, not structure, and therefore functions in the absence of an available structure. In this paper the SMERFS algorithm is systematically evaluated alongside other methods on a new large collection of protein-protein and protein-small molecule interface examples derived from the SNAPPI-DB database [24].

## Results and Discussion

### Comparison of SMERFS with MINER

SMERFS was compared to the tree-based method, MINER [25] for prediction on the SNAPPI-DB set. Table 1 shows the *ROC*_0.1 _scores for predictions of domain-small molecule, domain-domain and the combined interacting residue sets. Both methods were applied with their respective optimal parameter sets as determined by *ROC*_0.1 _score over 9 training sets.

**Table 1:** Comparison of Results for SMERFS and MINER. *ROC*_0.1 _scores for optimised parameters of SMERFS and MINER (window size, *W*, gap threshold, *C*_*g *_and scoring scheme), as tested in the blind data subset, separate from the training data. In the 'Combined' site type, protein and small molecule binding residues are pooled to form a single standard set. In the central scoring scheme, scores produced by SMERFS or MINER are applied to the central position of the window only, in 'max', all alignment positions take the score of the window covering them with the maximum score. For the 0–0.1 false positive rate range here, a random prediction would on average produce an area of 0.005.

Interaction Type	Scoring Method	Optimum Parameters			*ROC*_0.1 _score	Significance of difference
				
		*W*	*C*_*g*_	Scoring Scheme		
Small Molecule	SMERFS	9	1.00	max	0.0079	0.947
	MINER	1	0.85	central	0.0096	
Domain	SMERFS	9	1.00	max	0.0071	0.824
	MINER	3	0.75	central	0.0060	
Combined	SMERFS	9	1.00	max	0.0077	0.160
	MINER	3	0.65	max	0.0070	

SMERFS performed better than MINER in the prediction of domain-domain interactions, with the methods scoring 0.0071 and 0.0060, respectively, the converse was true in prediction of small molecule contacts. However, none of the differences were significant at the 0.05 confidence level. This result suggests that the additional complexity in construction of phylogenetic trees in MINER does not help improve accuracy.

### Conservation Measures

Optimal parameters were determined for each method on the SNAPPI-DB training set as described in Methods. Table 2 shows results for 14 conservation measures on the SNAPPI-DB blind test set. Results are shown both for normalised single-column scores (standard) and for optimal combinations of multi-position smoothing and gapped position removal (optimised). The SMERFS results from Table 1 are included for comparison, and in these rows the 'standard' result was derived from the matrices of single alignment positions, while 'optimised' indicates multi-column matrices.

**Table 2:** Results Comparison of 14 Conservation Measures and SMERFS. *ROC*_0.1 _scores for 14 conservation measures taken from [6], SMERFS ,and a random control. Results shown are for a blind testing set of Pfam families, employing optimised parameters derived from prior cross-validation. Each score was normalised to a 0–1 range for an alignment, and inverted when necessary so that 1 = max conserved. This score is shown in the table as 'Standard'. Results of an additional, 'optimised' form are also shown, smoothed over a window of *W *positions, and processed to remove column results with gap content above *C*_*g*_. The score difference column contains optimised – standard *ROC*_0.1 _scores.

Interaction Type	Conservation Measure	Reference	*ROC*_0.1 _score (standard)	Parameters		*ROC*_0.1 _score (optimised)	Score Difference
						
				*W*	*C*_*g*_		
Domain	Williamson	[28]	0.0087	3	1	0.0098	0.0011
	Kabat	[29]	0.0079	5	1	0.0088	0.0009
	Karlin	[31]	0.0074	7	1	0.0086	0.0012
	Sander	[36]	0.0078	3	1	0.0083	0.0005
	Valdar	[32]	0.0075	7	1	0.0081	0.0006
	Taylor	[33]	0.0070	9	1	0.0080	0.0010
	Zvelebil	[52]	0.0070	9	1	0.0079	0.0009
	*SMERFS*		*0.0056*	*9*	*1*	*0.0071*	0.0015
	Armon	[14]	0.0063	9	0.8	0.0067	0.0004
	Jores	[30]	0.0050	9	0.9	0.0064	0.0014
	Schneider	[36]	0.0049	5	0.6	0.0062	0.0013
	Gerstein	[35]	0.0049	5	0.6	0.0062	0.0013
	Thompson	[26]	0.0060	7	1	0.0062	0.0002
	Mirny	[34]	0.0059	9	0.7	0.0057	-0.0002
	Randomscore		0.0048	7	1	0.0052	0.0004
	Lancet	[27]	0.0008	1	0.3	0.0051	0.0043
Small Molecule	Williamson	[28]	0.0114	5	1	0.0122	0.0008
	Kabat	[29]	0.0128	3	1	0.0120	-0.0008
	Valdar	[32]	0.0120	1	1	0.0120	0.0000
	Taylor	[33]	0.0113	1	1	0.0113	0.0000
	Karlin	[31]	0.0121	9	1	0.0110	-0.0011
	Thompson	[26]	0.0073	1	0.9	0.0108	0.0035
	Zvelebil	[52]	0.0110	9	1	0.0108	-0.0002
	Armon	[14]	0.0107	1	1	0.0107	0.0000
	Schneider	[36]	0.0091	1	0.8	0.0102	0.0011
	Gerstein	[35]	0.0091	1	0.8	0.0102	0.0011
	Lancet	[27]	0.0014	1	0.2	0.0097	0.0083
	Sander	[36]	0.0091	3	1	0.0093	0.0002
	Jores	[30]	0.0090	9	0.8	0.0090	0.0000
	*SMERFS*		*0.0057*	*9*	*1*	*0.0079*	0.0022
	Mirny	[34]	0.0089	9	0.6	0.0078	-0.0011
	Randomscore		0.0049	7	1	0.0053	0.0004
Combined	Williamson	[28]	0.0107	5	1	0.0112	0.0005
	Kabat	[29]	0.0106	3	1	0.0108	0.0002
	Karlin	[31]	0.0099	7	1	0.0108	0.0009
	Valdar	[32]	0.0100	7	1	0.0102	0.0002
	Taylor	[33]	0.0093	9	1	0.0098	0.0005
	Zvelebil	[52]	0.0091	9	1	0.0097	0.0006
	Sander	[36]	0.0091	3	1	0.0096	0.0005
	Schneider	[36]	0.0064	5	0.5	0.0078	0.0014
	Gerstein	[35]	0.0064	5	0.5	0.0078	0.0014
	*SMERFS*		*0.0057*	*9*	*1*	*0.0077*	0.0020
	Armon	[14]	0.0083	9	0.8	0.0076	-0.0007
	Jores	[30]	0.0066	9	0.9	0.0074	0.0008
	Lancet	[27]	0.0009	1	0.2	0.0073	0.0064
	Thompson	[26]	0.0065	9	1	0.0068	0.0003
	Mirny	[34]	0.0074	9	0.7	0.0067	-0.0007
	Randomscore		0.0049	7	1	0.0053	0.0004

Performance results for optimised measures in Table 2 do not always match or exceed those of the standard form. This is because optimised parameters for all measures were derived on the SNAPPI-DB training set, which is distinct from the blind test set. For prediction of domain contacts, all methods are improved on parameter optimisation with the exception of *Mirny*. In the case of small molecule contacts however, only 7 of 14 conservation measures perform better in optimised than the standard form. For example, *Kabat *scored 0.0128 in its standard form compared to 0.0120 optimised. *Thompson *[26] and *Lancet *in contrast *were *improved by optimisation- *Thompson *was improved from 0.0073 to 0.0108 by removal of the most highly gapped positions, though the optimised 'window' size was 1 as in the standard form. The lack of universal improvement in results from parameter optimisation in the small molecule validation set may reflect a reduced level of dependency of small-molecule binding positions with their neighbours, compared to protein-binding positions.

All methods predicted small molecule contacts better than inter-domain contacts, as judged by *ROC*_0.1 _score, reflecting the increased difficulty in prediction of domain-domain contacts. Ranking of the different measures was largely conserved between domain-domain and domain-small molecule standard sets, with only minor re-ordering.

All measures achieved better scores than random, with the exception of *Lancet *[27] in domain-domain contact prediction, which scores 0.0051, compared to a random score of 0.0052.

The best performing measure for both domain-domain (*ROC*_0.1 _= 0.0098) and domain-small molecule (*ROC*_0.1 _= 0.0122) contact prediction was Williamson [28], a relative entropy measure incorporating physicochemical property scores as shown in Equation 1:

(1) $$Williamson=\sum_{i}^{K}p_{i}\ln\left(\frac{p_{i}}{{\bar{p}}_{i}}\right)$$

Where K = 9, for 9 physicochemical residue sets used, and *p*_*i *_is *f*_*i*_/*N *where *f*_*i *_is the frequency of residue set *i *in a column and *N *is the number of sequences. ${\bar{p}}_{i}$ is the mean value of *p*_*i *_over all alignment columns. Interestingly, the next best score, in both domain-domain and domain-small molecule contact prediction tasks was given by the simple *Kabat *measure [29] illustrated in Equation 2:

(2) $$Kabat=\frac{k}{n_{1}}\times N$$

Where *k *is the number of amino acid types present in the alignment column, *n*_*i *_is the the number of times the most commonly occurring amino acid occurs in the column, and *N *is the number of sequences in the alignment. The difference in score between *Williamson *and *Kabat *was not significant at the p = 0.05 confidence level. The measure *Jores *[30] is only slightly more complex than *Kabat*, employing a consideration of residue pairs present at a given position. However, the results in Table 2 shows that this addition has not improved performance, and *Kabat *consistently outperformed *Jores *with p-values from McNemar's test of 1.9 × 10^-28^, 1.9 × 10^-16 ^and 4.1 × 10^-31 ^in prediction of domain, small-molecule and combined interacting positions, respectively. It may be that the extreme simplicity of *Kabat *is it's strength, rendering it robust to noise compared to other measures, and this feature is lost in *Jores*. Alternatively the alignments in the data set may contain too few sequences for the relative benefits of more complex measures to be fully demonstrated.

Other measures which gave consistently high scores in prediction of both types of interface were the mutation data score *Karlin *[31], the weighted sum-of-pairs score *Valdar *[32], and the stereochemical property score, *Taylor *[33], all of which incorporate amino acid properties into their function.

Table 2 also highlights the worst performing measures, *Mirny *[34] and *Lancet *[27]. This is interesting since *Mirny *is closely related to *Williamson*, as shown in Equation 3:

(3) $$Mirny=\sum_{i}^{K}p_{i}\ln p_{i}$$

Notation is as Equation 1, except *K *= 6. Unlike *Williamson*, Equation 3 does not normalise scores according to the frequencies of residue types in the alignment.

The worst performing measure in the domain-domain interaction prediction task, *Lancet *[27] is defined by Equation 4:

(4) $$Lancet=\sum_{a}^{K}\sum_{b}^{K}\frac{p_{a}p_{b}}{M(a,b)}$$

Where *p*_*a *_is the fractional frequency of amino acid *a *in the aligned column, *K *represents the alphabet of amino acids, and *M*(*a, b*) is a substitution matrix such as BLOSUM62. *Lancet *was noted in [6] to suffer from idiosyncrasies related to placement of *M*(*a, b*) as a denominator, it may be that this is at the root of its poor performance here.

The equivalent *Gerstein *[35] and *Schneider *[36] measures also performed badly. Similarly to *Williamson *they are based on entropy in a column of the alignment, but unlike *Williamson *and *Valdar*, they do not incorporate any consideration of physicochemical properties.

In summary, the best performing conservation measures tend to incorporate terms to normalise for the character of the alignment in question, as well as the relationships between residues according to their physicochemical properties. In *Williamson *[28] normalisation is in terms of the characteristic residue type frequencies of an alignment, in *Valdar *[32] it is the degree of sequence redundancy present. Poorly performing measures lack one or more of these features. *Kabat *is an anomaly in these results, lacking most of the features present in other successful measures. This may be a consequence of simplicity, endowing the measure with a strong resistance to noise that outweighs some of its shortcomings, but may also be an artefact of the dataset.

Since *Williamson *gave the best performance, it is used in the remainder of this paper as representative of conservation measures in comparison with other techniques.

Recently, Capra and Singh have published a related study [7] of conservation as a method of predicting functional residues. The authors employed a set of active site residues as standards; since such residues are not employed in the current study, direct comparisons cannot be made. However two other categories are used by the authors: 'ligand distance' and 'homolog protein interface', which are roughly equivalent to the 'small molecule' and 'domain' interacting residues of the current study.

Two measures are shared by the present study and that of Capra and Singh: the *Mirny *property entropy score and the *Karlin *sum-of-pairs score. Comparison based on these measures shows that *ROC*_0.1 _scores for un-optimised measures are higher in the current study, as shown in Table 2. In Capra and Singh's work, *Karlin *scores 0.0086 and 0.0069 for small molecule and domain interactions, respectively, while *Mirny *scores 0.0049 and 0.0037. This is likely to reflect differences in the origins of the validation data used; the validation sets here may be more complete in annotation, being derived from SNAPPI-DB. Capra and Singh did not examine the *Williamson *entropy of properties score [28], which this study has shown to be superior to *Mirny*. Had they done so, the indications from this study are that a performance closer to their best-performing 'Jensen-Shannon divergence' may have been produced.

### Comparison of Conservation Measures and SMERFS

Table 2 also shows results for SMERFS, including those from single alignment columns rather than the optimum over different window sizes. The optimised form shows a large improvement over the single column 'standard' form. The top conservation measure, *Williamson*, out-performed SMERFS in both domain-domain (SMERFS score = 0.0071) and domain-small molecule (SMERFS score = 0.0079) contact prediction. The significance of difference by McNemar's test are 1.1 × 10^-15 ^and 3.1 × 10^-12^, respectively. Since the increased complexity of SMERFS did not appear to have produced a corresponding increase in performance on our benchmark, the cause of the superior performance of simple measures such as *Williamson *[28] was examined.

### Characterisation of the Difference Between SMERFS and Williamson Conservation

As outlined in the introduction and in Figure 1, conservation and phylogeny based techniques such as SMERFS aim to identify different types of functional residues. Accordingly, the predictions by *Williamson *and SMERFS were examined by considering the number of predictions in common between the methods, by breaking down results by complex type on the BW05 set and by classifying the predictions into accessibility classes. Finally, a qualitative comparison of the methods was performed for the amino transferases.

#### Comparison of True Positive Sets

The overlap of true positive sets from SMERFS and the best performing conservation measure was assessed on the SNAPPI-DB data set. True positive sets were created by applying a threshold to the 0–1 position scores generated by each method chosen to coincide with a false positive rate of 0.1.

Table 3 illustrates the difference and overlap in the predictions of the true positive sets from SMERFS and *Williamson *in predictions on the three subsets of the SNAPPI-DB data: small molecule, domain-domain, and combined. The overlap of true positive sets between methods was small in comparison to the true positive set of either method. For example in small molecule contact prediction, the overlap was by 921 positions, compared to 4771 true positives from *Williamson*, and 4436 for SMERFS. This illustrates the fundamentally different types of position predicted by each method.

**Table 3:** Overlap between true positive (TP) sets produced by Williamson and SMERFS. True positive sets were created by applying a threshold corresponding to a false positive rate of 0.1 in both measures, as detailed in Methods.

Site Type	SMERFS only	Overlap	*Williamson *only
Domain-Small Molecule	3515	921	3850
Domain-domain	6989	1995	8204
Combined	9945	2183	8990

Despite the superior results produced by *Williamson *when considering ROC areas in the SNAPPI-DB dataset, choosing the threshold for both methods via fixed false positive rate cost produced similar performance in terms of absolute true positives (TP). Higher ROC areas indicate a higher mean true positive rate, defined as TP/(TP+FN). Given a similar number of true positives, this indicates an elevated number of false negatives for SMERFS compared to conservation. This was presumably due to SMERFS failing to detect the most highly conserved regions since they lack any pair-wise similarity pattern.

#### Construction of a Hybrid Classifier

Since predictions from SMERFS and *Williamson *appear complementary, a predictor was developed that combined the two approaches within a Bayesian framework. Classifiers were trained in a cross-validation scheme, recording the probability of an interacting position conditional on combinations of scores from SMERFS and *Williamson*. However, validation of these classifiers did not give a consistent improvement on the best-performing of either SMERFS or *Williamson*.

#### Results in the BW05 data set: Different types of interface require different approaches

**Figure 2:**
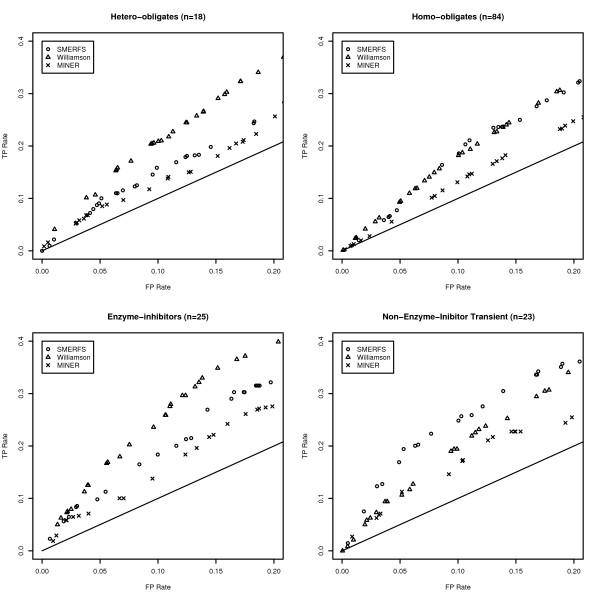
**Partial ROC Plots from the BW05 Data**. Partial ROC plots illustrating the difference in method performance in each of the four categories of interface that comprise the Bradford data set. The straight line shown represents the ratio of TP rate to FP rate expected from a randomly generated measure.

The SNAPPI-DB set is an extensive set of interacting positions that were produced automatically from structural data. The BW05 data set [37] contains complexes of manually verified biological significance, separated into 4 subsets of different character. Figure 2 illustrates the performance of SMERFS relative to other measures in the most stringent portion of the ROC curve to a false positive rate of 0.2. The significance of the difference was assessed at a false positive rate of 0.1 by McNemar's test and displayed in Table 4. Comparison with Table 2 reveals an improved performance for both SMERFS and *Williamson *on the BW05 dataset. For example, the lowest SMERFS score is found in the hetero-obligate subset in Table 4 with a *ROC*_0.1 _of 0.0084; the comparable domain-domain value in Table 2 is 0.0071. As a more refined dataset than SNAPPI-DB, this set posseses a lower proportion of interacting positions, but those that *are *present are reliable. This may mean that a greater proportion of negatives assigned by good prediction measures are likely to be assigned as true negatives (TN), reducing false positive rates (FP/FP+TN), and thereby increasing *ROC*_0.1 _scores.

**Table 4:** Results of ROC analysis of SMERFS and Williamson methods applied to the BW05 data.

Complex Type	*ROC*_0.1_		Significance
		
	SMERFS	*Williamson*	
Hetero-obligate	0.0084	0.0118	0.0059
Homo-obligate	0.0089	0.0095	0.83
Enzyme-inhibitor	0.01044	0.0142	0.011
NEIT	0.0152	0.0107	0.025

Table 4 also highlights the performance differences between methods, and between different data subsets. SMERFS performed particularly well in the NEIT (non-enzyme inhibitor transient with a score of 0.0152, outperforming *Williamson*'s score of 0.0107 with a significance of 0.025. Structures in this category include murine gamma herpesvirus cyclin complexed to human cdk2 (PDB code 1F5Q) and a guanine nucleotide exchange factor for Ras-like GTPases (PDB code 1bkd). *Williamson *outperformed SMERFS in enzyme-inhibitor (e.g. porcine pancreatic trypsin, PDB code 1AVW) and hetero-obligate complexes (e.g. Calcineurin-like phosphoesterase PDB 1TCO), while the difference in performance was negligible for the homo-obligate complexes. The performance differences highlight the necessity of choosing a prediction method appropriate to the system of interest. The more successful prediction by *Williamson *for enzyme-inhibitor and hetero-obligate contacts suggests a high level of conservation in the positions that mediate them. Conservation methods should therefore be the primary choice in prediction of this type of interface. Conversely, transient complexes seem to involve alignment positions with weaker conservation that inhibit the predictive ability of *Williamson*, but enhance that of SMERFS.

#### Surface Accessibility of Predicted Positions

Since the definition of 'functional residue' employed here is a residue that mediates contact with other molecules, the most successful measures may be expected to predict accessible positions preferentially. Surface accessibility was compared between the predicted residue sets of different methods in the BW05 set. Relative surface accessibilities for residues were calculated with the program NAccess (unpublished, S. Hubbard and J. Thornton 1992–6), and their distributions compared between methods. All other structural entities except the domain of interest were removed from the PDB file prior to this analysis, so that otherwise accessible positions were not obscured by interacting entities.

**Figure 3:**
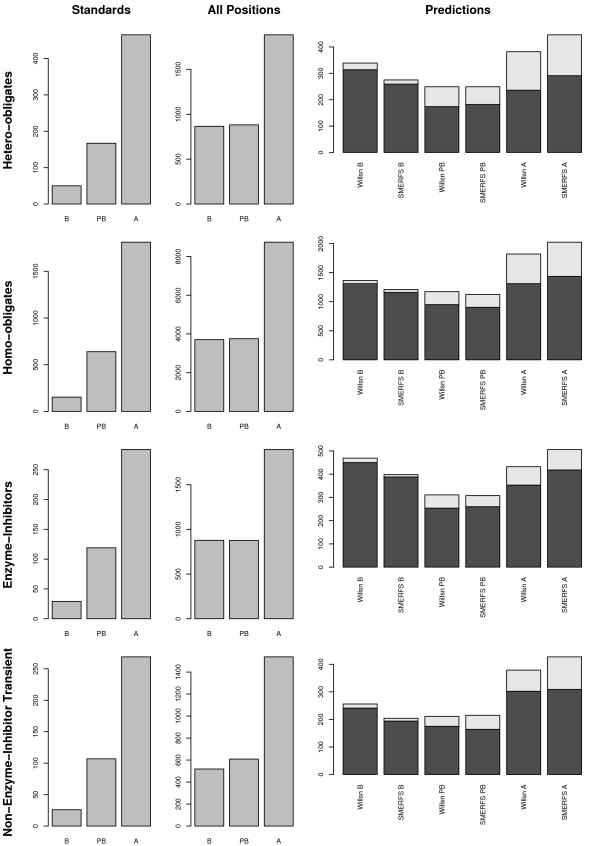
**Surface Accessibility of Domain Residues**. Illustration of surface accessibility of positions associated with functional residue prediction in the four different interface subtypes of the BW05 validation data [37]. Distributions of relative solvent accessibility (RSA) are shown in divided into 3 bins: buried (B, 0 ≤ *RSA *< 5), partly buried (PB, 5 ≤ *RSA *< 25), and accessible (A, 25 ≤ *RSA *< 100). Of the three columns of panels, the far left (labeled 'Interacting Positions' represents all positions found interacting in the BW05 dataset. Centre panels illustrate positions over the domain as a whole, while right-hand panels are derived from SMERFS or *Williamson *predictions. The lighter 'caps' on bars in the 'Predictions' column represent the portion that corresponds to interactions in the BW05 set. Rows describe the sub-types of hetero-obligates, homo-obligates, enzyme-inhibitors and non-enzyme inhibitor transient (NEIT).

Figure 3 illustrates the surface accessibility of the predicted residue sets from SMERFS and *Williamson*, relative to that of the residues in the Bradford standard set, and in the domain as a whole. Accessibility is shown in 3 categories of percent relative solvent accessibility, *RSA*: buried (B, 0 ≤ *RSA *< 5), partially buried (PB, 5 ≤ *RSA *< 25), and accessible (A, 25 ≤ *RSA *= 100). Accessibility of BW05 interacting positions is shown in the left panels. As might be expected from known interacting positions, a greater proportion of these positions are accessible, compared to the total set of domain residues shown in the centre panels (labelled 'All Domain Positions'). The far right panels show distributions of accessibility for the predicted sets, with lighter colored sections representing the portion of predictions corresponding to interacting positions of the BW05 data. Compared with the 'All Domain Positions' plots, predicted residue sets have a high buried (B) content. This may in part be due to prediction of positions of core structural importance, and in part due to buried positions associated with interfaces. The distributions produced by the two methods are, however, different, with SMERFS consistently producing the more accessible predictions. The difference is significant (as assessed by the Mann-whitney rank-sum test for unpaired data) in all sets at better than p = 0.01. This suggests SMERFS predicted fewer core structural positions in this analysis set, and more surface residues more likely to form interactions with other molecules. To guage the impact this accessibility might have on comparative accuracy between methods, the analyses described in section was repeated, limited to positions of > 25% accessibility. However this did not provide an improvement in results (data not shown), possibly due to the sparse (albeit high-confidence) nature of the BW05 dataset.

#### Case Study: Aminotransferases

**Figure 4:**
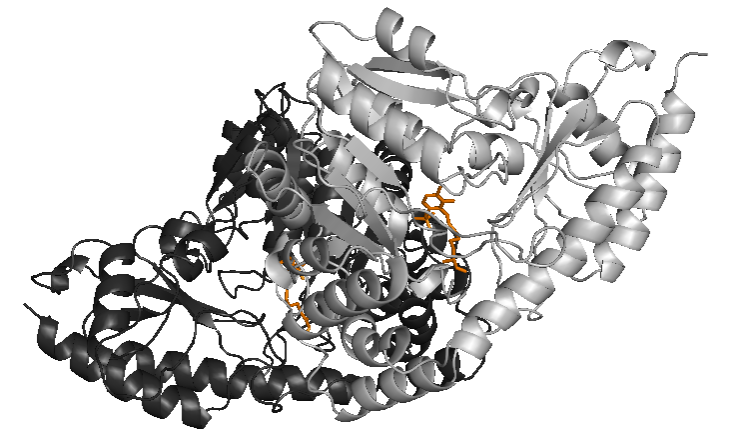
**Complex Structure of PDB Structure **1BW0**, Representing Pfam Family PF00155**. Illustration of the dimer structure for Pfam family PF00155 (amino transferase class I and II) in the *Trypanosoma Cruzi *structure (PDB code 1BM0, [38]). The complex comprises 2 chains, chain A is shown in dark gray, chain B in lighter grey. The two PLP cofactor molecules are shown in orange, and each has contacts with both chains.

The relative strengths and weaknesses of conservation vs phylogeny based approaches is illustrated for Pfam family PF00155, 'amino transferase class I and II', taken from the homo-obligate subset of the BW05 data. This family was selected as an example due to its single domain structure, and the presence of a large amount of information on both protein-protein and protein-small molecule interactions. Family members function as dimers and bind a pyridoxal phosphate cofactor, as shown in an example structure from *Trypanosoma Cruzi *(Figure 4[38]).

**Figure 5:**
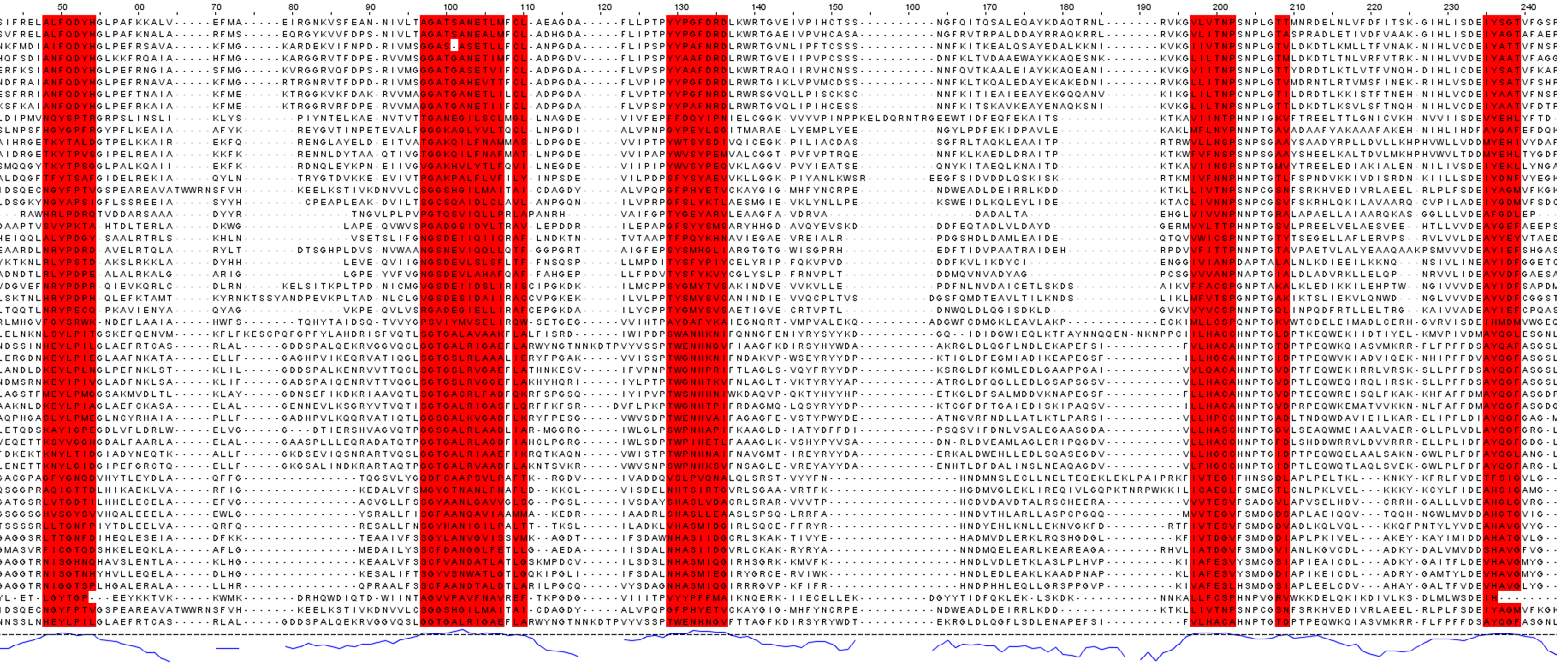
PF00155 seed alignment excerpt with SMERFS-predicted positions shown in red.

**Figure 6:**
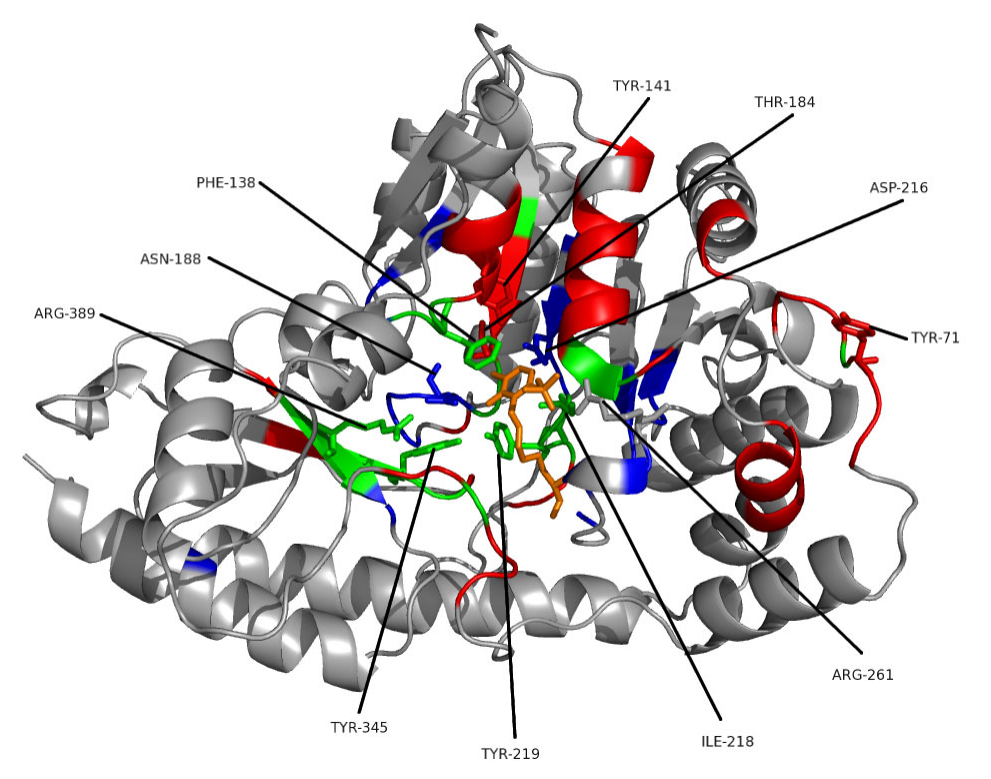
**Ligand-binding Positions of Tyrosine Aminotransferase of Trypanosoma Cruzi**. One chain of the crystal structure of tyrosine aminotransferase from *Trypanosoma Cruzi *(PDB code 1BW0). Results of a conservation-based measure (Williamson, in blue) are shown compared to the phylogeny-based SMERFS (in red). Positions predicted by both techniques are shown in green, the PLP cofactor in orange. Protein regions in stick representation and labelled are those important for cofactor binding, as described in the text.

Figure 5 shows part of the seed alignment for Pfam family PF00155, with red-highlighted regions showing columns predicted as functionally important by SMERFS. Figure 6 shows the locations of putative functional positions as predicted by SMERFS and *Williamson *within one chain of the complex. Figure 6 also shows, in stick representation, some of the important cofactor and substrate-binding residues described in Blankenfeldt et al [38]. There are a number of conserved positions, which were detected by *Williamson *and not SMERFS, as expected due to the lack of family structure in these regions. These include Lys253 and Asp216 which serve to orientate the phosphate-binding residue Arg261, and Asn188 that hydrogen bonds to PLP's phenolic oxygen. Interestingly, Arg261 itself, which forms a salt bridge with the phosphate group of PLP, was not predicted as functional by either method due to a gap present in sequences of one subfamily of the alignment. Examination of the literature for these sequences (for example KBL ECOLI, [39]), reveals structural evidence that these enzymes are still capable of binding pyridoxal phosphate, but must do so by means not requiring a positive charge in this region.

Other important positions around the cofactor binding site were predicted by SMERFS and not *Williamson*. Tyr141 and Thr184 are known to stabilise Asp216 by hydrogen bonding, and Tyrosine 71 hydrogen bonds to the cofactor. However these positions vary across the family as a whole, since hydrogen bonds can be supplied by a large variety of residues. As a consequence, these positions do not display sufficient conservation to be ranked highly by conservation.

**Figure 7:**
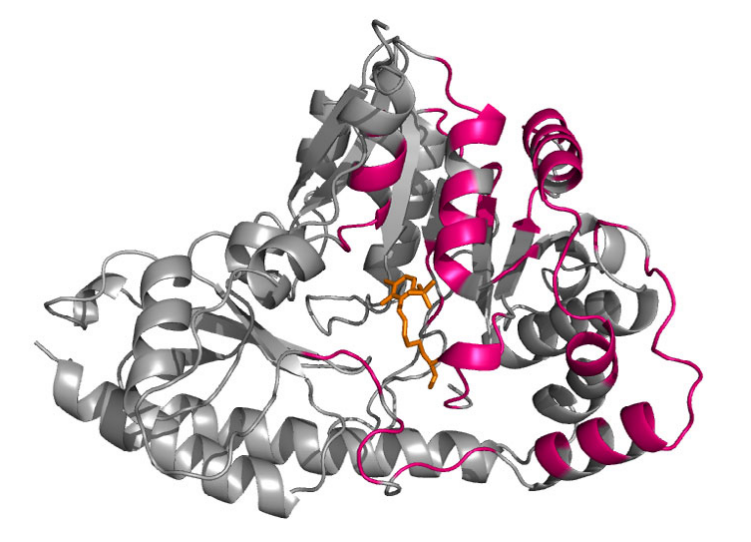
Structure as Figure 6, highlighting the domain-domain interface in pink.

The main advantage of SMERFS over *Williamson *evident in this example is the ability to predict a larger number of the protein-protein contacts. This can be seen by comparing the predicted positions shown in Figure 6 with the chain-chain contacts highlighted for comparison in Figure 7. Of 87 protein-protein contacting positions annotated to the PF00155 alignment from the BW05 data set, SMERFS predicts 32, compared to just 10 from *Williamson*. These positions, while exhibiting patterns in the pair-wise sequence relationships that are detectable by SMERFS, are not detected as easily by conservation.

A number of positions were predicted successfully by both methods. Tyr 219 binds with Asn188 to PLP's phenolic oxygen. Tyr345 is known to stabilise Asn188 and Tyr219. A stacking interaction provided by Phe138 and a contact with Ile218 form other important PLP interactions, and were predicted by both methods. These positions are not invariant in the alignment, but conserve properties, for example of aromaticity or aliphaticity; this supplies both subfamily structure detectable by SMERFS and property conservation detectable by *Williamson*. Interestingly, Arg389, a crucial and absolutely conserved position in the substrate-binding region is also predicted by SMERFS. Despite the lack of family structure displayed at this position, SMERFS assigns it as positive due to patterns present in the surrounding positions. The successful and exclusive prediction by SMERFS of many of the positions comprising the hydrogen bonding network at a ligand binding site illustrates its utility. Performance in protein-protein interaction prediction is also much better for SMERFS than *Williamson*, and while not generalisable beyond this example, this shows that algorithms such as SMERFS that detect specificity defining positions can sometimes succeed where conservation cannot.

The aminotransferase class I and II sequence family has features that make it particularly amenable to analyses of this type. The sequences are single-domain, without the more complex evolutionary history present in multi-domain proteins. The sequences are sufficiently divergent for a subfamily structure to develop, without being so diverse as to introduce a large number of gaps. Additionally, the associated 3D structures for the family are well annotated, with large numbers of both domain-domain and domain-small molecule interacting positions known. This reduces the number of times a correctly predicted position is not recognised as such, a problem in larger, less well annotated sequence families.

## Conclusion

The work presented here has shown that SMERFS produces sets of putative functional positions in multiple sequence alignments fundamentally different from those of conservation measures. For this reason conservation measures and phylogeny-aware methods such as SMERFS should be considered as complementary tools. The data suggest that if alignment positions involved in the core function of a protein, for example catalysis, are the target of a study, relatively simple conservations measures remain the most useful tool. If less critical positions, perhaps responsible for defining sequence subfamiliy specificity, are the target, then methods such as SMERFS may be of use. Finally, SMERFS has been shown to predict many more surface positions than conservation, reducing the possibility of confusing signals from positions of core structural rather than functional significance.

The complementary nature of conservation measures and those that seek specificity-defining positions are strongly suggestive of a combined approach. However, attempts made as part of this study to produce a hybrid classifier based on a rigorous probabilistic approach have failed. This may be because the relative importance of specificity-defining positions is different for differing complex types. For this reason a potentially useful approach would be to construct different combined classifiers for complexes of different types. The BW05 set contains too little data to produce accurate probabilities of sites for given score pairs based on observations, but a larger set of this type available in future might produce improved results. If method combination is not feasible, a simpler approach would be to determine those characteristics that make an alignment more amenable to one type of analysis or another, and select measures accordingly. Despite the work described here and elsewhere in prediction of functional residues, it remains true that the accuracy of functional residue prediction techniques falls short of that necessary for true utility in the laboratory. There are multiple likely causes of this, and therefore multiple potential solutions.

The first is the definition of 'functional residue' employed by studies such as this. Protein residues may be closely implicated in the hydrogen bonding networks that stabilise an interacting site without being directly involved in the interaction. This was demonstrated in the aminotransferase case study above, but only direct interactions formed part of the validation data sets. Perceived performance of prediction methods might improve if residues peripheral to interacting sites are included in analyses, though such information is harder to derive automatically from structural data alone.

From a pure sequence-based perspective, difficulties result from the variable level of divergence present in protein sequence families. Families with either insufficient sequences, or too little divergence between homologues confound analysis since too little evolutionary history is evident to discern true conservation. Too many sequences or too much divergence, and patterns may be lost in noise or obscured by gaps. It may be possible to tackle this issue by careful choice of sequences for analysis, and estimation of likely accuracy given the sequence data available.

Another key factor in an analysis like this is the fact that the meaning of 'functional position' is dependent on the protein family in question. This study has shown that the performance of methods is variable depending on the type of interaction under consideration. A study that sub-divided interactions into a larger number of classes would be likely to reveal further differences between methods.

## Methods

### Validation Data

Two structurally derived sets of standards were employed in the development and validation of the methods described here: a large automatically generated set of sites (the SNAPPI-DB set), and a smaller, hand curated dataset derived from the work of Bradford and Westhead [37] (the BW05 set).

#### The SNAPPI-DB Set

1652 seed alignments were extracted from Pfam [40,41], chosen for the availability of associated data on domain-domain and domain-small molecule contacts in SNAPPI-DB [24]. SNAPPI-DB is a high-performance object-oriented database derived from the European Bioinformatics Institute's MSD database [42]. The MSD is a data warehouse that includes all data in the PDB [43], but with substantial additional derived data, including 'likely biological units'. SNAPPI-DB contains atom-level information on interactions between protein domains from SCOP [44], CATH [45] and Pfam [40]. The presence of contacts due to crystal packing artefacts is limited by the use of PQS [46].

Interacting residues were defined as all those with 1 or more atoms within 0.5 Å plus the Van der Waals radius of the small molecule or protein domain binding partner. An additional requirement for the definition of a domain-domain interface was that at least 10 residues were involved in the interaction. Interface residues from all associated structures were then annotated to the parent Pfam family. A further standard set was constructed that combined both domain-domain and domain-small molecule interacting residues (the 'combined set') as a test of real-world accuracy when all types of sites would be predicted simultaneously.

Four alignments of > 1000 sequences were excluded, since processing these by some methods was too time-consuming. 191 alignments with fewer than 5 sequences were removed due a requirement of MINER [25]. This reduced the set to 1,457 families. In this final set, 32,339 columns over 1,229 families (8%) were annotated as small-molecule contacts, while 68,323 columns in 1,300 families (25%) were annotated as involved in contacts with other protein domains.

The test alignments ranged from 20 to 792 sequences (mean 81), and from 27 to 1,812 columns in length (mean 235). Average pair-wise percentage identity varied from 14–85% (mean 34). Pair-wise percentage identity is the number of identical positions divided by the length of the shortest sequence, multiplied by 100.

### The BW05 Set

The SNAPPI-DB set provides a large set of domain-domain and domain-small molecule interfaces; however it does not provide finer-grained classification of the interfaces. For this reason methods were also assessed on the smaller manually curated set of 180 PDB chains derived by Bradford and Westhead [37]. This set defines four types of interaction: hetero-obligates, homo-obligates, enzyme inhibitors and non-enzyme-inhibitor transient (NEIT). Obligate interfaces were defined as those within a stable rather than transient quaternary structure. The annotated regions of 144 of these chains could be mapped to 163 PFAM seed alignments. Of these, 13 were rejected since they had less than 5 sequences and 2 for sizes in excess of 1000 sequences. This resulted in a final analysis set of 148 Pfam seed alignments referred to as the BW05 set (Table 5).

**Table 5:** Description of the BW05 data set. The data comprising and derived from the BW05 dataset [37]. Total is the total number of unique Pfam [40] domains or chains involved. Since there are Pfam domains that occur in more than one subset, this is not the sum over all subsets. NEIT = 'non-enzyme-inhibitor transient'.

Data Type	Homo-obligate	Hetero-obligate	Enzyme-inhibitor	NEIT	Total
PDB Chains	87	27	36	30	180
Chain with Pfam domains	78	24	31	27	160
Total Pfam domains covering annotated regions	89	21	29	27	163
Pfam domains with seed alignments in 5–1000 sequence range	84	18	25	23	148

### SMERFS Algorithm

**Figure 8:**
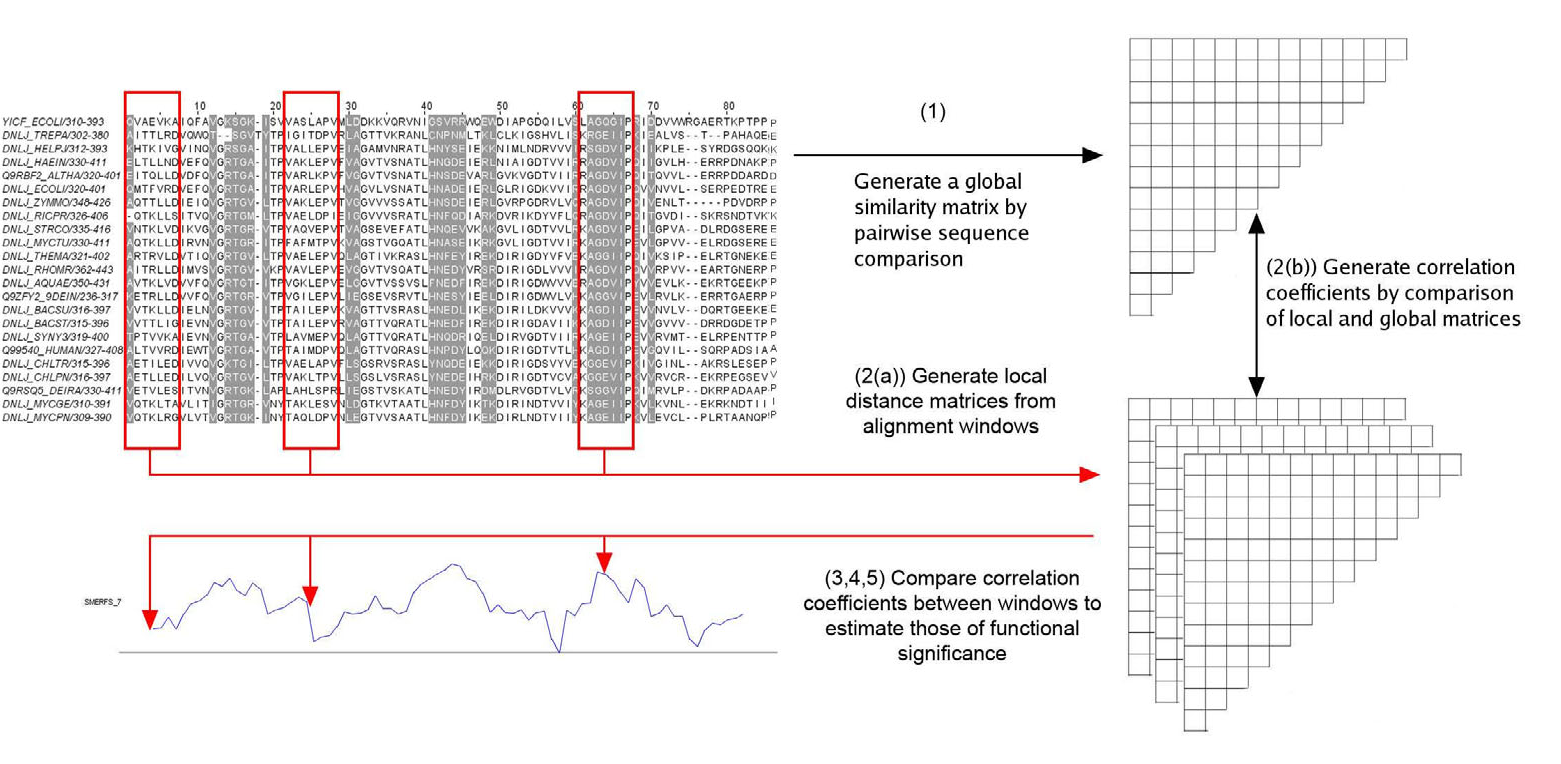
**Illustration of the SMERFS Algorithm**. Illustration of the SMERFS algorithm, showing Pfam family PF03120 with a trace resulting from SMERFS run with a window size of 7. Red highlighting on the alignment shows the known locations of interactions with other domains. See text for details.

The SMERFS algorithm is intermediate in philosophy to those of TreeDet [21] and MINER [18] and compares local to global similarity matrices over windows on an alignment. Figure 8 summarises the SMERFS algorithm which proceeds in the following steps:

1. Construct a global similarity matrix, *G*, over all *N *sequences in an alignment *A*, of length *L*. The matrix *G *contains *N*(*N *- 1)/2 similarity values (*S*_*ab*_) for all unique sequence pairs.

The similarity between each pair of sequences is estimated by summing pair-wise inter-residue BLOSUM 62 scores over all aligned positions between all pairs of sequences. ProtDist is often employed for this purpose [47], but is too slow to be used for high-throughput investigations of large numbers of sequences required here. Fortunately, there is a close correlation between pair-wise distances generated by ProtDist and those derived by BLOSUM matrices (data not shown).

2. Take a window of width *W *columns and move along the alignment in steps of *k*, where 1 ≤ *k *<*M *and *M *is the length of the alignment. For each central window position, *i*:

(a) Generate a pair-wise similarity matrix, *L*_*i *_over all subsequences present in the window.

(b) Calculate the Pearson correlation coefficient, *r*_*i *_between *L*_*i *_and *G*.

3. Optionally process scores so that that an individual position is assigned the score of the highest-scoring window of which it is a part. This is the 'maximum scoring scheme'.

4. Reject positions with a gap content above *C*_*g *_%

5. Adjust for the diversity of the current alignment by normalising the correlation coefficients to vary between 0 and 1 over the alignment. The result of this process is a graph that can be viewed with an alignment as shown in Figure 8.

6. Positions with *rnormi*_*i *_above a given threshold *T *are predicted as functionally significant.

SMERFS is implemented as a set of Perl objects and exploits the PDL library of C-linked modules [48].

### Conservation Measures

For comparison of SMERFS with measures that consider conservation without consideration of pair-wise relationships, 14 of the conservation measures reviewed by Valdar [6] were implemented. In addition, a measure was used as a control where each position was assigned a random value in the range 0–1. For each measure both a single-column, 'standard' form without further modification and an 'optimised' form was generated. To calculate the optimised form:

1. Sum the scores over an odd number of positions, *W*, in the range 1–9. Apply the mean to the central position.

2. Reject positions with a gap content above *C*_*g *_%

### ROC Analysis

Positive sets were derived from the results of each method by selection of the top-ranked positions, and assigned as 'true' (TP) if they were present in the validation data, false otherwise. ROC curves were created by plotting the true positive (TP) rate (fraction of correctly classified positives, or TP/(TP + FN))against the false positive (FP) rate (fraction of incorrectly classified negatives, or FP/(FP + TN)) at each of a progressively larger section of the top ranked positions.

This assessment was stringent, since only residues in direct contact with other entities were assigned as TP. The results reported should therefore be considered a lower bound on accuracy.

#### ROC area

The area under a ROC-curve provides a measure of accuracy over a range of coverage. This allows comparison without the need to choose a point on the curve corresponding to a single threshold placed on range of score produced by a method. In this work the ROC curve up to a false positive rate of 0.1 was considered in order to favour methods that produce high TP rate results at low FP rates. This area is referred to as the *ROC*_0.1 _score. A perfect prediction will produce a *ROC*_0.1 _score of (0.1 × 1) = 0.1, while a random prediction will result in a *ROC*_0.1 _score of (0.1^2^/2) = 0.005.

#### Assessment of Significance

Standard, non-parametric methods are available to assign significance to the difference between ROC areas [49,50]; however these are only applicable to full ROC curves. Estimates of significance of the difference between ROC curves were thus obtained by use of McNemar's test [51]. Differences between the number of true positives produced in all methods at a fixed false positive rate of 0.1 were assessed. If *TP*_*a *_and *TP*_*b *_are the number of true positives *specific *to sets A and B respectively, then McNemar's test produces a chi-squared statistic as shown in Equation 5:

(5) $$\chi^{2}=\frac{{\left(TP_{a}-TP_{b}\right)}^{2}}{TP_{a}+TP_{b}}$$

The *p *value associated with this statistic was derived from standard tables.

#### Parameter Optimisation

Family alignments from the SNAPPI-DB data set were divided into 10 subsets (7 of 146 sequences, 3 of 145). One subset was put aside as a final blind test, and optimal method parameters determined by 9-way cross-validation on the remaining sets. SMERFS, MINER, and conservation measures were optimised to select an odd numbered window length in the range 1–9, and a gap threshold *C*_*g *_in the range 0.05–1 (increments of 0.05). SMERFS and MINER were additionally optimised to determine if the 'maximum' scoring scheme should be applied. Final measures of performance for each method were then determined by the *ROC*_0.1 _score for the blind set.

## Authors' contributions

JM carried out algorithm development and all analyses described. EJ developed the SNAPPI-DB database and supplied derived information for use in this study. GJB conceived and supervised the study and helped to write the manuscript. All authors have read and approved the final manuscript.

## Note in proof

During the proofing of this manuscript, we were contacted by Capra and Singh [7], who had compared their JSD method to the Williamson conservation measure on their data set. They reported un-windowed ROC_0.1 values of 0.033 and 0.008 for enzyme-ligand and protein-protein interfaces respectively for their method, compared to 0.025 and 0.008 for Williamson. We have carried out a preliminary analysis of our SNAPPI-DB dataset using code supplied by Capra and Singh. In our hands, the only improvement over Williamson was for small molecule interactions, but this was insignificant.
